# Public Health Centers' Training Session Programs to Develop Programs on Infection Control Practices for Multidrug-Resistant Organisms in Hospitals in Kawaguchi City, Japan

**DOI:** 10.7759/cureus.48178

**Published:** 2023-11-02

**Authors:** Ayako Nakayama, Ichiro Yamaguchi, Koji Okamoto, Shigefumi Maesaki

**Affiliations:** 1 Administration Department, Kawaguchi Public Health Center, Saitama, JPN; 2 Department of Environmental Health, National Institute of Public Health, Saitama, JPN; 3 Department of Infectious Disease and Infection Control, Saitama Medical University, Saitama, JPN

**Keywords:** providing information, evidence including numerical effects, training sessions, public health center, infection control, multidrug-resistant organisms

## Abstract

Introduction

The Kawaguchi City Public Health Center (PHC) conducted training sessions focusing on infection control practices on multidrug-resistant organisms (MDROs) for 19 hospitals and eight affiliated clinics (AFs) with beds in June 2022. Issues with infection control programs were identified via a survey implemented following the training sessions. These included providing feedback on infection control policies for MDROs, hand hygiene compliance programs (HHCPs), environmental cleaning (EC), and training sessions programs that hospitals or AFs with beds (hospitals) intended to implement in the future or develop (to be developed). We planned to examine whether the PHC training sessions programs have an effect on the development of hospital infection control programs designed to address these issues. The purpose of this study is to clarify the training session program provided by the Kawaguchi City PHC, which was effective in developing hospital infection control programs based on the results of the survey conducted after the training session.

Methods

In June 2023, a second training session that offered information on infection control practices was completed for 30 hospitals. This was followed by sending a questionnaire. We examined infection control programs to be developed and analyzed associations with the first learned information by training session (the first learned information).

Results

Twenty-four hospitals responded to the survey with a response rate of 80.0%. Half the respondents (12, 50.0%) had prepared for the infection control policy on carbapenem-resistant Enterobacteriaceae (CRE), 11 hospitals (45.8%) had provided feedback on HHC, and four (16.7%) planned to conduct feedback on HHC. HHCPs were planned to be developed by 19 hospitals (79.2%), EC by five hospitals (20.8%), training session by 12 hospitals (50.0%), and screening of MDROs upon hospital admission (AS) by nine hospitals (37.5%).

The first learned information, “the prevention of healthcare-associated infections and cost savings by implementing cleaning bundles (the effects of cleaning bundles),” was identified by 10 hospitals (41.7%), and “specific programs on providing feedback effective for developing hand hygiene compliance (specific feedback)” was learned by eight hospitals (33.3%).

The first learned information regarding specific feedback was significantly associated with HHCPs to be developed (p = 0.044). The first learned information on the effects of cleaning bundles was significantly associated with HHCPs and HHC feedback to be developed (p = 0.023, 0.034). The training session programs were not significantly connected to EC, training session, or AS to be developed.

Conclusions

Infection control programs to be developed were linked to the provision of information on numerical effects by implementing specific feedback and cleaning bundles. We suggest that the PHC should develop infection control programs for the hospitals and provide training sessions, including numerical effects.

## Introduction

A significant issue in public health is that of multidrug-resistant organisms (MDROs). The World Health Organization’s World Health Assembly in May 2015 adopted a worldwide action plan on antimicrobial resistance (AMR), which represents the general view that AMR could pose a serious threat to human health if no action is taken against MDROs. In Japan, the government created the National Action Plan (NAP) on AMR (2016-2020) and the NAP on AMR (2023-2027) in April 2023 and has initiated concerted efforts to combat AMR. Regarding MDROs, Japan has a lower incidence of carbapenemase-producing Enterobacteriaceae (CPE), a type of carbapenem-resistant Enterobacteriaceae (CRE), at 0.19 cases per 100,000 population in 2020, which is lower than that of England [1-3]. Nevertheless, since 2020, about 2,000 cases of CRE per year have been notified [4].

In the United States, local public health departments are visiting healthcare facilities with high rates of CRE to communicate with healthcare professionals and prevent CRE infections [5]. The surveillance reported by 25% of Japan’s hospitals reveals that CRE was identified in 51.2% of medical institutions, and the number of identified CRE cases was over 9,000 [6]. Therefore, the public health center (PHC) should advice on preventing the spread of CRE infection, not only for the hospitals in which CRE has been notified but also for those in which it has never been notified. There are few infection preventionists (IPs) and no university hospitals in the core city of Kawaguchi, which is located near Tokyo and has a population of roughly 600,000. Therefore, it can be challenging for the hospital personnel in Kawaguchi City (Kawaguchi) to get assistance from experts on infection control (IC).There were 20 hospitals and 12 affiliated clinics (AFs) with beds in Kawaguchi in 2022. The routine visits to these facilities by the PHC, stipulated by law, had revealed that most of these facilities had not prepared for IC policies for MDROs (e.g., CRE) and had not developed hand hygiene (HH) compliance programs (CPs).

In June 2022, the Kawaguchi PHC provided training sessions (TSs) to enhance IC programs to 19 hospitals and eight AFs with beds to address the lack of information in such situations, specifically focusing on MDROs [7]. The results of a survey carried out after the TS showed that nine facilities (39.1%) had provided feedback on HH compliance and five facilities (21.7%) had prepared for IC policies for CRE [7]. Environmental cleaning (EC) of patients’ rooms with MDROs that hospitals or AFs with beds (hospitals) intended to develop and implement in the future were identified by 10 (43.5%) hospitals, and TSs were identified by six (26.1%) hospitals [7]. These findings lead us to conclude that additional information must be provided to target the development of the IC issues listed earlier. Local public health authorities that educate facilities on MDROs conducted the survey on IC practice [8] and had not reported whether the information provided by them had any effect on IC programs by the hospitals. Therefore, we concluded that we need to examine if TS programs by the PHC have any effect on the development of IC programs by the hospitals.

To address the IC issues identified by the survey in 2022, we conducted a second TS for 20 hospitals and 10 AFs with beds in Kawaguchi in June 2023. The purpose of this study is to clarify the TS programs provided by the PHC that are effective in developing the IC programs by the hospitals by examining the survey results conducted after the TSs [9].

This article was previously presented as a meeting poster at the 2022 Nihon Kousyuueisei Gakkai Annual Meeting on October 8, 2022.

## Materials and methods

There were 20 hospitals and 11 AFs with beds in Kawaguchi in 2023. We emailed all of these facilities about the TS programs provided by the Kawaguchi PHC, and 20 hospitals and 10 AFs with beds in Kawaguchi applied for TSs. In June 2023, the Kawaguchi PHC conducted a TS focusing on HHCPs and IC practices on MDROs and the evidence to enhance IC programs for these 20 hospitals and 10 AFs with beds via a web conference system facilitated by a public health physician (Table 1). The TS participants were medical doctors, pharmacists, nurses, and hospital clinical laboratory technicians. We e-mailed a questionnaire to 20 hospitals and 10 AFs with beds, requested their permission to participate in the study, and indicated that the respondents’ personal information would be removed from the questionnaire results before they were made public. Out of the total, a response rate of 80.0% was obtained from18 hospitals and six AFs with beds [9].

**Table 1:** Training session programs on IC and prevention of MDROs by the Kawaguchi City Public Health Center IC: infection control; MDROs: multidrug-resistant organisms; EC: environmental cleaning; ICU: intensive care unit; CPE: carbapenemase-producing Enterobacteriaceae; MRSA: methicillin-resistant *Staphylococcus aureus*; MDRA: multidrug-resistant *Acinetobacter*

Description	Training session programs
Hand hygiene compliance programs	Performance feedback may increase adherence to hand hygiene practices and reduce infection and colonization rates.
	A ward-level hand hygiene compliance program can be successful in improving hand hygiene rates.
	Feedback on the mean compliance rate for the last seven days should be provided.
	Individual compliance rates were publicly posted in designated areas.
	Simulation training sessions were effective for hand hygiene compliance programs.
	Each ward should be responsible for setting its own hand hygiene rate goal.
	Hand hygiene champions, doctors, and nurses should lead hand hygiene compliance programs.
	Targets of 10–20% hand hygiene improvement were set.
Evidence on enhanced EC	Admission to an ICU room previously occupied by a patient with MDROs was a risk factor for acquisition of the bacteria by subsequent room occupants.
	Despite implementing enhanced IC, including contact precautions, the transmission of CPE through indirect ward and hospital contact did not decrease.
	Genomic sequencing of isolates from patients revealed links to previous CPE outbreak strains from two years prior.
	Implementing the cleaning bundle resulted in a decrease in MDROs and cost savings.
Evidence to remove dust and EC that the hospitals should conduct	We recommend that the hospitals remove dust in relevant areas by mopping, in reference to IC policies by university hospitals.
	During the outbreak, the single MRSA strain was widespread in the dust in the ward environment.
	The MDRA outbreak strains were isolated from the dust of respiratory ventilators and continuous veno-venous hemofiltration.
Evidence to clean sink and drains and EC that the hospitals should conduct	Do not keep medications or patient care supplies on surfaces that are within 1m of sinks.
	A splash barrier should be used.
	The sink has a basin depth of at least 240 mm.
	Covers should be put on sink drains.
	CPE plasmids from the patient were linked to those from two sinks in the patient’s room.
Importance and evidence on monitoring and feedback of EC	The possibility of transmission through inanimate objects could not be ruled out in the MDRO outbreak.
	Monitoring of and feedback from EC is recommended.
	Enhanced EC practices, including monitoring and feedback, and hiring new personnel can reduce MDROs infection rates.
	EC monitoring was conducted using a fluorescent marking gel when exposed to ultraviolet light, and feedback was provided to the EC staff and the manager in the previous study in which the health-associated infection decreased.
Evidence on holding training session	A higher percentage of staff with IC training was associated with a decrease in MRSA infections.
	IC training within the last three years was associated with higher knowledge scores regarding MDROs.

The following topics were covered by the responses to the hospitals’ questionnaire: preparedness for IC policy on MDROs (IC policy), HHCP, HHCP that hospitals intended to implement in the future or develop (to be developed), IC policy on EC that the hospitals intended to prepare (to be prepared), IC programs to be developed, TS on MDROs, TS that hospitals intended to conduct (to be conducted), the first learned information from TS (the first learned information), and useful information or activities for developing IC (useful information) [9].

Variables excluding the IC policy were all summarized using the number of hospitals. The IC policy was summarized for each MDRO individually. Multiple and single regression analysis were used to determine the relationship between IC programs to be developed and the first learned information. Furthermore, a correlation analysis was carried out to determine the relationships between IC programs to be developed and the first learned information and IC programs to be developed and useful information. This study used Office Excel (Microsoft, WA, USA) for basic data aggregation and multiple and single regression analyses and correlation analysis.

The role of the Japanese PHC is to ensure safety in the hospitals by guiding them to develop their IC programs [10,11]. The role of the hospitals is to develop their IC programs and practices [11]. In this study, we aimed to provide effective information to develop the IC programs by the hospitals in the jurisdiction, and we analyzed the survey results that were defined as city activities in Kawaguchi stipulated by law and examined them based on research report recommendations and best practices.

This study involves no invasive procedures (e.g., drawing blood, collecting samples, or asking traumatic questions) and is not intended to use human subjects. Hence, the ethics committee did not have to approve this study.

## Results

**Figure 1:**
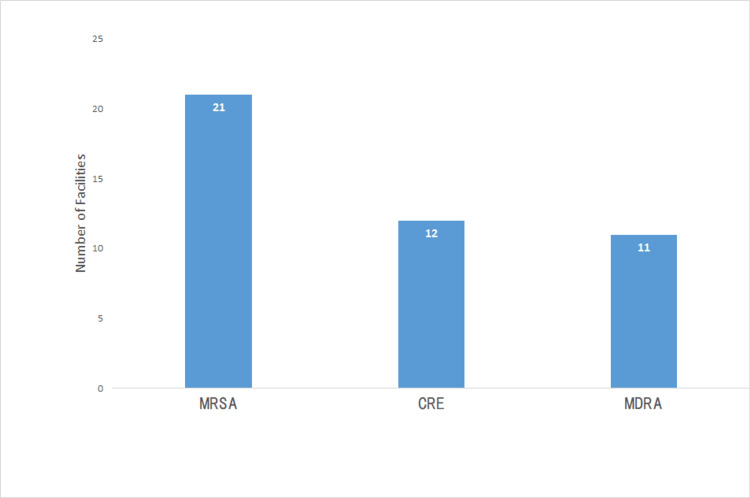
Infection control policies on MDROs (n = 24) MDROs: multidrug-resistant organisms; MRSA: methicillin-resistant *Staphylococcus aureus*; CRE: carbapenem-resistant Enterobacteriaceae; MDRA: multidrug-resistant* Acinetobacter*. Data are presented as N.

**Figure 2:**
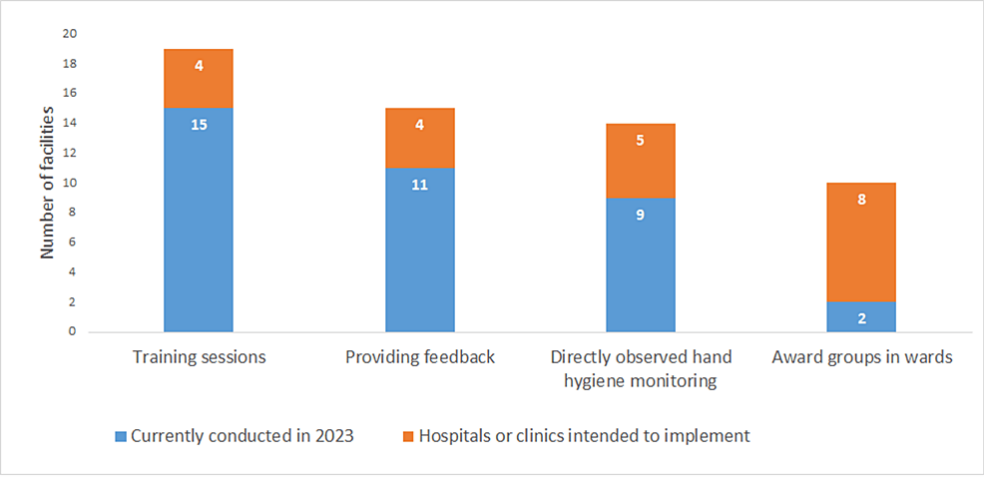
Hand hygiene compliance programs and those that hospitals intended to implement in the future or develop (n=24) Award groups in wards: award groups in wards and departments whose hand hygiene compliance rates are high. Data are presented as N.

**Figure 3:**
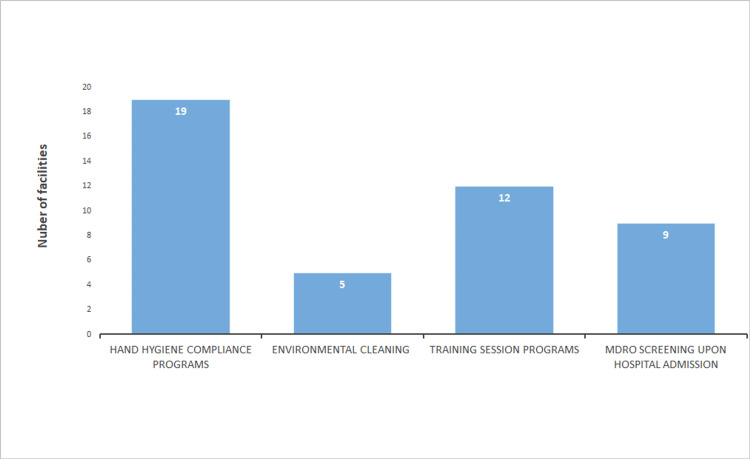
Infection control programs that hospitals intended to implement in the future or develop MDRO: multidrug-resistant organism Data are presented as N.

**Figure 4:**
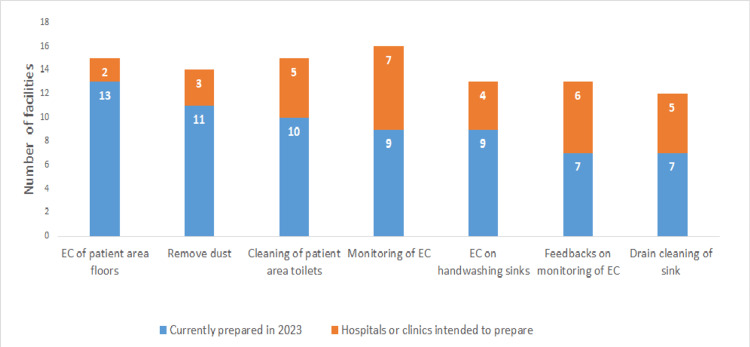
Infection control policy on EC and those that hospitals intended to prepare (n = 24) EC: environmental cleaning Data are presented as N.

**Figure 5:**
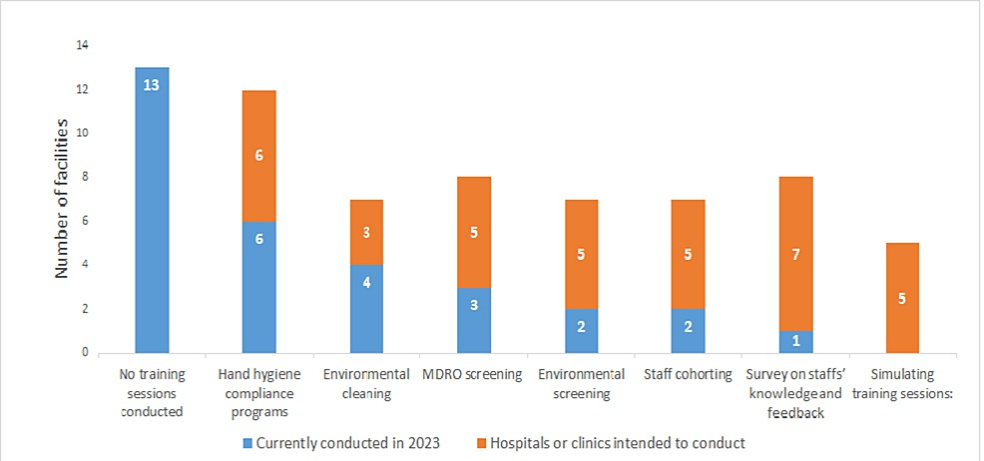
Training session on MDROs and those that hospitals intended to conduct (n = 24) MDRO: multidrug-resistant organism Data are presented as N.

Half of the hospitals (12, 50.0%) had prepared for the IC policy on CRE and 11 hospitals (45.8%) for multidrug-resistant *Acinetobacter* (MDRA) (Figure 1). Eleven hospitals (45.8%) had provided feedback on HH compliance (Figure 2). Four hospitals (16.7%) planned to conduct feedback on HH compliance (Figure 2). HHCPs were planned to be developed by 19 hospitals (79.2%), EC by five hospitals (20.8%), TSs on MDROs by 12 hospitals (50.0%), and screening of MDROs upon hospital admission (AS) by nine hospitals (37.5%; Figure 3). Nine hospitals (37.5%) had prepared for EC monitoring, and seven hospitals (29.2%) had prepared for feedback on EC monitoring in their IC policy (Figure 4). Thirteen hospitals (54.2%; Figure 5) had not conducted TSs on MDROs. Five hospitals (25.0%) intended to conduct TSs on staff cohorting (SC) (Figure 5). None of the hospitals had conducted TSs on simulated IC measures, and five hospitals (25.0%) intended to conduct them (Figure 5).

**Figure 6:**
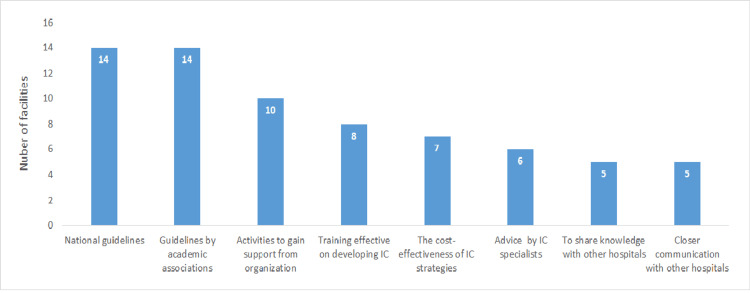
Useful information and activities on developing IC by the hospitals (n = 24) IC: infection control Data are presented as N.

The first learned information included the prevention of healthcare-associated infections (HAIs) and cost savings by implementing cleaning bundles (the effects of cleaning bundles) by 10 hospitals (41.7%), specific programs on providing feedback effective for developing HH compliance (specific feedback) by eight hospitals (33.3%), and the effect of TSs on IC, such as a decrease in MDROs associated with TSs (the effect of TSs) by three hospitals (12.5%; Table 2). “National guidelines” were identified by 14 hospitals (58.3%) as useful information, “guidelines by academic associations” were identified by 14 hospitals (58.3%) as such, and “activities to gain support from organizations” (activities for organizations) were identified by 10 hospitals (41.7%) as such (Figure 6).

**Table 2:** First learned information from training sessions (n = 24) MRSA: methicillin-resistant* Staphylococcus aureus*; MDRA: multidrug-resistant *Acinetobacter*; EC: environmental cleaning; IC: infection control; CPE: carbapenemase-producing Enterobacteriaceae Data are presented as N (%).

Description	Number of facilities	Percentage (%)
MRSA strain was widespread in the dust in the ward environment in the outbreak.	12	50.0
MDRA outbreak strains were isolated from the dust of respiratory ventilators.	11	45.8
Implementing the cleaning bundles resulted in preventing healthcare-associated infections and cost savings.	10	41.7
Enhanced EC practices were effective on IC.	9	37.5
Specific feedback was effective for hand hygiene compliance programs.	8	33.3
Despite implementing enhanced IC measures, the transmission of CPE through indirect ward and hospital contact did not decrease.	8	33.3
Sequencing of CPE isolates from patients revealed links to previous outbreak strains.	8	33.3
Simulation training sessions were effective for hand hygiene compliance programs.	7	29.2
Feedback was effective for hand hygiene compliance programs.	6	25.0
The TS programs were effective on IC.	3	12.5

The first learned information “specific feedback” was significantly associated with HHCP to be developed (p = 0.044; Table 3). The effects of cleaning bundles were significantly related to HHCP, assessed HH compliance and provided feedback (providing feedback) to be developed (p = 0.023, 0.034, 0.034, 0.008; Table 3). The following IC programs to be developed were not related to the first learned information: EC, TS, and AS. The effects of TSs were significantly associated with simulating TSs on IC measures and TSs on SC to be conducted (p = 0.038, 0.038; Table 3).

**Table 3:** Association between the first learned information from training sessions and IC programs that hospitals intended to implement in the future or develop (n = 24) IC: infection control; RC: regression coefficient; CI: confidence interval; HH: hand hygiene; CPE: carbapenemase-producing Enterobacteriaceae; EC: environmental cleaning; MDROs: multidrug-resistant organisms Results by multiple regression analysis and single regression analysis. Statistical significance determined as P ≤ 0.05.

IC that hospitals intend to implement in the future or develop	First learned information from training sessions	Multiple regression analysis			Single regression analysis		
		Standard partial RC	p-value	95% CI	RC	p-value	95% CI
HH compliance programs	Specific feedback effective for HH compliance programs	0.43	0.044	0.01–0.84	0.31	0.081	−0.04–0.67
	CPE linked to previous outbreak	0.07	0.729	−0.35–0.49	0.13	0.499	−0.25–0.50
	Cleaning bundles' effects	0.39	0.023	0.06–0.73	0.36	0.034	0.03–0.69
	Effect of training session programs on IC	−0.25	0.455	−0.95–0.44	0.24	0.364	−0.29–0.77
Assess HH compliance and provide feedback to the staffs	Specific feedback effective for HH compliance programs	−0.04	0.850	−0.42–0.35	−0.06	0.713	−0.41–0.29
	CPE linked to previous outbreak	0.23	0.233	−0.16–0.62	0.31	0.056	−0.01–0.63
	Cleaning bundles effects	0.34	0.034	0.03–0.65	0.40	0.008	0.12–0.68
	Effect of training session programs for IC	−0.05	0.863	−0.70–0.59	0.19	0.430	−0.30–0.68
EC of the MDRO patients’ room	Specific feedback effective for HH compliance programs	0.01	0.961	−0.50–0.53	0.06	0.736	−0.32–0.44
	CPE linked to previous outbreak	0.03	0.915	−0.49–0.55	0.06	0.763	−0.36–0.49
	Cleaning bundles effects	−0.03	0.853	−0.45–0.38	0.16	0.426	−0.24–0.56
	Effect of training session programs for IC	0.12	0.768	−0.74–0.99	0.14	0.588	−0.40–0.68
Training session programs on MDROs	Specific feedback effective for HH compliance programs	0.29	0.306	−0.29–0.88	0.19	0.409	−0.27–0.65
	CPE linked to previous outbreak	0.15	0.590	−0.43–0.74	0.19	0.409	−0.27–0.65
	Cleaning bundles effects	0.35	0.138	−0.12–0.82	0.34	0.106	−0.08–0.76
	Effect of training session programs for IC	−0.25	0.600	−1.23–0.73	0.19	0.557	−0.47–0.85
Simulating training sessions on IC measures	Specific feedback effective for HH compliance programs	−0.17	0.444	−0.63–0.29	0.06	0.736	−0.32–0.44
	CPE linked to previous outbreak	0.00	0.990	−0.46–0.47	0.25	0.169	−0.11–0.61
	Cleaning bundles effects	0.07	0.716	−0.30–0.43	0.16	0.372	−0.20–0.51
	Effect of training session programs for IC	0.63	0.101	−0.14–1.40	0.52	0.038	0.03–1.01
Training sessions on staff cohorting	Specific feedback effective for HH compliance programs	−0.17	0.444	−0.63–0.29	0.06	0.736	−0.32–0.44
	CPE linked to previous outbreak	0.00	0.990	−0.46–0.47	0.25	0.169	−0.11–0.61
	Cleaning bundles effects	0.07	0.716	−0.30–0.43	0.16	0.372	−0.20–0.51
	Effect of training session programs for IC	0.63	0.101	−0.14–1.40	0.52	0.038	0.03–1.01
MDRO screening upon hospital admission	Specific feedback effective for HH compliance programs	−0.40	0.126	−0.93–0.12	−0.19	0.393	−0.63–0.26
	CPE linked to previous outbreak	−0.13	0.617	−0.66–0.40	0.19	0.393	−0.26–0.63
	Cleaning bundles effects	0.32	0.135	−0.11–0.74	0.39	0.058	−0.01–0.79
	Effect of training session programs for IC	0.65	0.142	−0.24–1.53	0.33	0.285	−0.30–0.96

The correlation coefficients for the first learned information (specific feedback, CPE patients’ strain linked to previous outbreaks, and effects by the cleaning bundle and effects of TSs) were 0.062, −0.060, 0.535, 0.298, 0.535, and 0.192 (Table 4). No correlation exists between HHCP and AS and activities for organization, with correlation coefficients of 0.017 and 0.044, respectively (Table 5).

**Table 4:** Correlation between the first learned information for the hospitals and IC programs that hospitals intended to implement in the future or develop (n = 24) IC: infection control; HH: hand hygiene; CPE: carbapenemase-producing Enterobacteriaceae; MDROs: multidrug-resistant organisms

Variables		Correlation coefficient
The first learned information for the hospitals		
Specific feedback effective for HH compliance programs	CPE linked to previous outbreak	0.062
Specific feedback effective for HH compliance programs	Cleaning bundles effects	−0.060
Specific feedback effective for HH compliance programs	Effect of training session programs on IC	0.535
CPE linked to previous outbreak	The cleaning bundles effects	0.298
CPE linked to previous outbreak	Effect of training session programs on IC	0.535
Cleaning bundles effects	Effect of training session programs on IC	0.192
IC that hospitals intend to implement in the future or develop		
HH compliance programs	Assess HH compliance and provide feedback to the staff	0.229
HH compliance programs	EC of the MDRO-identified patients’ rooms	0.011
HH compliance programs	Training sessions programs on MDROs	0.308
HH compliance programs	Simulating training sessions on IC measures	0.011
HH compliance programs	Training sessions on staff cohorting	0.011
HH compliance programs	MDROs screening upon hospital admission	0.185
Assess HH compliance and provide feedback to the staff	EC of the MDRO-identified patients’ room	0.046
Assess HH compliance and provide feedback to the staff	Training sessions programs on MDROs	0.447
Assess HH compliance and provide feedback to the staff	Simulating training sessions on IC measures	0.321
Assess HH compliance and provide feedback to the staff	Training sessions on staff cohorting	0.451
Assess HH compliance and provide feedback to the staff	MDROs screening upon hospital admission	0.346
EC of the MDRO-identified patients’ rooms	Training sessions programs on MDROs	0.308
EC of the MDRO-identified patients’ rooms	Simulating training sessions on IC measures	0.242
EC of the MDRO-identified patients’ rooms	Training sessions on staff cohorting	0.495
EC of the MDRO-identified patients’ rooms	MDROs screening upon hospital admission	0.026
Training sessions programs on MDROs	Simulating training sessions on IC measures	0.513
Training sessions programs on MDROs	Training sessions on staff cohorting	0.513
Training sessions programs on MDROs	MDROs screening upon hospital admission	0.430
Simulating training sessions on IC measures	Training sessions on staff cohorting	0.747
Simulating training sessions on IC measures	MDROs screening upon hospital admission	0.662
Training sessions on staff cohorting	MDROs screening upon hospital admission	0.450

**Table 5:** Correlation between IC programs that hospitals intended to implement in the future or develop and useful information and activities on developing IC by the hospitals (n = 24) IC: infection control; HH: hand hygiene; EC: environmental cleaning; MDROs: multidrug-resistant organisms

IC programs to be developed	Useful information and activities on developing IC by the hospitals	Correlation coefficient
HH compliance programs	Activities to gain support from organization by other hospitals	0.017
HH compliance programs	Evaluation of the cost-effectiveness of infection control strategies by other hospitals	0.120
HH compliance programs	To be provided advice and visiting the hospitals by infection control specialists	−0.415
Assess HH compliance and provide feedback to the staffs	Activities to gain support from organization by other hospitals	0.076
Assess HH compliance and provide feedback to the staffs	Evaluation of the cost-effectiveness of infection control strategies by other hospitals	0.205
Assess HH compliance and provide feedback to the staffs	To be provided advice and visiting the hospitals by infection control specialists	-1.326E-16
EC of the MDRO-identified patients’ rooms	Activities to gain support from organization by other hospitals	0.399
EC of the MDRO-identified patients’ rooms	Evaluation of the cost-effectiveness of infection control strategies by other hospitals	0.339
EC of the MDRO-identified patients’ rooms	To be provided advice and visiting the hospitals by infection control specialists	0.415
Training sessions programs on MDROs	Activities to gain support from organization by other hospitals	0.338
Training sessions programs on MDROs	Evaluation of the cost-effectiveness of infection control strategies by other hospitals	0.123
Training sessions programs on MDROs	To be provided advice and visiting the hospitals by infection control specialists	0.192
MDRO screening upon hospital admission	Activities to gain support from organization by other hospitals	0.044
MDRO screening upon hospital admission	Evaluation of the cost-effectiveness of infection control strategies by other hospitals	0.311
MDRO screening upon hospital admission	To be provided advice and visiting the hospitals by infection control specialists	0.348

## Discussion

TS programs to encourage the development of IC programs by the hospitals

In Japan, there are no national guidelines for IC on MDROs including HH compliance. Meanwhile, the importance of monitoring HH practices is emphasized by the WHO [12]. Directly observed monitoring and feedback for HH might not contribute to the development of HH compliance [13]. Given this information, we provided information on specific feedback useful for developing HH compliance (Table 1), which was not related to providing feedback to be developed (Table 3). We suggest that the hospitals in which feedback was not provided at the time of the survey and that intended to provide feedback might have known about specific feedback effective in developing HH compliance. Moreover, we suggest that the effects of the cleaning bundle, which includes enhanced HHCP, resulted in strong association with HHCP and provided feedback to be developed (Table 3). We propose that providing information regarding the effects of IC practices could be useful in helping hospitals develop HHCP. Therefore, the public health authorities including the PHC (public health authorities) and IC specialists who provide the hospitals with advice on IC (IC specialists) need to provide them.

In Japan, 87.4% of the hospital ECs are performed by co-signed businesses (CBs) [14]. We suggest that hospital personnel could not anticipate improved EC from the CB worker if the hospital personnel had learned the evidence to enhance EC, resulting in EC being developed by few hospitals and TS programs not being significantly associated with EC to be developed (Table 3). The WHO stated that hospitals' providing educational programs for cleaning staff is crucial for appropriate EC [12]. Hospitals and CBs created teams to develop EC, and their activities included EC that was directly observed by IC staff. These activities resulted in EC by businesses reflecting the intentions of hospitals [15]. Based on this analysis, we recommend that the public health authorities and IC specialists provide information including activities to develop EC by the CB and guide the hospitals to enhance EC. The lack of knowledge on EC and inappropriate IC written policy on EC by the hospitals may contribute to the inappropriate EC by the CB in Japan [16]. Therefore, IC policy on EC and those to be prepared by other hospitals (Figure 4) might contribute to developing EC programs by the hospitals. The public health authorities and IC specialists need to provide them to increase the knowledge of the hospitals on EC.

Moreover, we suggest that the hospitals could not have expected that enhanced EC can prevent the spread of infections of MDROs, after they had learned the evidence that the identified patient was linked to a CPE outbreak two years prior (Table 1). Consequently, EC to be developed might not have been significantly associated with TS programs (Table 3). The hospitals are recommended to develop their acceptable EC after they examine their capability for implementing EC [17]. We suggest that the public health authorities and IC specialists might need to provide the hospitals with these recommendations to guide them to start developing EC programs.

We provided the hospitals with the effect of TSs and no specific effective TS programs (Table 1). We propose that the lack of specific effective TS programs [18] might contribute to not answering it to be the first learned information by the hospitals that were provided with insufficient information. Therefore, the public health authorities and IC specialists may need to provide specific TS programs that have some effects on TSs to be conducted. In Japan, there are no national guidelines for TSs on MDROs. Training of staff regarding MDROs is critical for the implementation of successful IC practices, as mentioned in the WHO guidelines [12]. Nevertheless, appropriate TSs on IC are challenging for the IP [19], and the sources of infection prevention information are limited, such as web-based training and reading the published literature [20]. The public health authorities and IC specialists might contribute to support the IP by examining the TS program and providing effective information on the TSs on MDROs to be conducted.

The WHO recommends that screening for asymptomatic CRE colonization should be based on patient risk assessment [12]. Moreover, SC and AS with a history of CPE infection or colonization or foreign hospital stay decreased CPE acquisitions [21], indicating that hospitals should examine the implementation of AS. We suggest that the absence of TSs on the effect of AS might contribute to TS programs that are not significantly associated with AS being developed. Therefore, the public health authorities and IC specialists need to provide TSs on the effect of AS to the hospitals [21]. In Japan, the annual cost of AS for 245 cases was approximately 1,400 USD (191,000 yen) [22]. Our recommendation is that hospitals be given information on the cost of AS so that they can conduct AS while demonstrating the cost burden. The AS of MDROs was impeded by a difficulty in identifying high-risk patients [23]. The public health authorities and IC specialists need to provide the hospitals with information on specific targets of AS [21] and guide them to conduct risk assessment on such.

Association between the organizational support and IC programs to be developed and addressing the barrier to implementing IC programs

The importance of organizational support is emphasized by IPs [24], as this encourages hospital staff to apply what they learn in IC training in their workplace [25]. Based on these findings, we had expected that IC to be developed was correlated with activities for the organization and not expected no correlation between HHCPs and providing feedback and AS and activities for organization (Table 5). To obtain appropriate support from hospital leadership, the IPs must demonstrate the cost and cost-effectiveness of IC practices with which the hospital leadership is not familiar [26]. Based on these findings, it might be suggested that the public health authorities and IC specialists may encourage the hospitals to develop their IC programs by providing information on the cost-effectiveness of implementing IC. Moreover, the public health authorities and IC specialists may need to collect information on activities to gain the support of hospital organizations (Figure 6) and provide the hospitals with them. Champions who lead the implementation of the best practices on IC can change the work environment and address the issues of organizational barriers [27]. The public health authorities and IC specialists need to provide the information that awarded groups in wards (Figure 2) or personnel contributed to the development of IC programs.

Providing information on numerical effects to promote developing IC programs

In this study, the TS programs (specific feedback effective for HHCPs, preventing HAI and cost savings by implementing the cleaning bundles, and the effect of TSs on IC, such as a decrease in MDROs) were associated with HHCPs and providing feedback to be developed and simulating TSs and TSs on SC to be conducted (Table 3). These TS programs also featured numerical effects of IC practices, including HH compliance, decrease in MDROs, and cost savings (numerical effects). It can be suggested that providing numerical effects may be crucial to helping hospitals develop IC programs. Therefore, we propose that the public health authorities and IC specialists encourage hospitals to develop AS, whose development was not associated with TS programs, by providing evidence, including numerical effects.

The structural indicator of the IC programs by the hospitals is that infection prevention programs should be reviewed [27]. Therefore, we need to conduct a survey on preparedness for IC policies on MDROs and EC (Figures 1, 4), HHCPs, and TSs on MDROs that are currently conducted (Figures 2, 5) to examine the effect of TSs.

Japanese national policy on IC and the importance of this study from a viewpoint of national and international policies on IC

In Japan, public health physicians have not been well trained on IC practices, and the Ministry of Health, Labor, and Welfare of Japan has indicated the policy that IC specialists who mainly study in the universities (IC specialists in the universities) take the lead in addressing the issues on IC practices in the hospitals in coordination with the PHC [28]. In Japan, there were 46 IC networks in which the IC specialists in the universities supervised the IC practices in the region [29]. Meanwhile, visiting the hospitals and providing advice by the IC specialists in the universities were conducted by 54.3% of these networks [29]. Moreover, in the area in Japan where there are few IC specialists in the universities, it is challenging for the PHC to obtain sufficient information to solve the issues under its jurisdiction. Therefore, providing the effective TS programs on IC programs in this study could develop the IC programs by the hospitals in the jurisdiction where the IC policy by the IC specialists in the universities is not covered.

In the United States, state and local health departments need to educate the hospitals on MDROs, with the support of the Centers for Disease Control and Prevention [30]. Local public health authorities communicated with the hospitals about IC [5] and conducted the survey on IC programs [8]. We could not find educational programs by the local public health authorities addressing the issues with IC programs. We suggest that with the issues in the jurisdiction where the state or local public health authorities do not address the specific issues, the IC programs might not be developed appropriately. Therefore, TS programs that develop programs on MDROs revealed by this study might contribute to improving the education by the state or local public health authorities.

Strengths and limitations of this study

In this study, we found that hospitals could develop their own IC programs when the PHC provided evidence on specific feedback and the effects of cleaning bundles and the effect of TSs on IC with numerical effects. We also suggest that the public health authorities and IC specialists need to provide the hospitals with the information on activities to gain the support of hospital organizations for developing HHCPs and AS and the cost-effectiveness of IC practices. Moreover, we suggest that the public health authorities and IC specialists need to provide information that awarded groups or personnel have contributed to the development of IC programs to address the issues of organizational barriers. We indicate that IC policies on EC by other hospitals need to be provided in developing EC by the CBs, which might be the barrier to the enhanced EC. The survey response rate in this study was relatively high (80.0%). This enabled us to collect information on IC programs to be developed by hospitals throughout the city. We anticipate that collecting specific effective information on IC programs from this study will not only help hospitals in other districts to develop IC programs but will also strengthen the PHC’s support for hospitals with regard to IC.

This study has several limitations. First, the information collected represents only 24 hospitals in Kawaguchi and may not fully represent IC programs in other regions. In addition, only a limited number of hospitals were covered by the PHC’s policy of providing information on hospitals’ IC practices. Moreover, TS programs not related to EC to be developed might not be applicable in other districts where the CB does not carry out the EC. Therefore, TS programs related to EC to be developed should be examined in other districts where the CB does not carry out the EC to examine the applicability. Furthermore, we did not examine preparedness for IC policy on MDROs and EC (Figures 1, 4), HHCPs, and TSs on MDROs that are currently conducted (Figures 2, 5), which are the indicators to examine the effect of this study. We must, therefore, acknowledge that the findings and programs implemented in this study might not be directly applicable to other districts or healthcare settings.

However, it is anticipated that the program implemented in this study, which aimed to provide effective information for developing IC programs, could serve as a model to be examined and adopted by other districts. The generalizability and effectiveness of the strategies utilized in this study will be determined by further investigations and evaluations in different settings.

## Conclusions

This study should be examined in other districts where CBs do not carry out the EC, and the effect of the study should be examined. However, we can conclude that TS programs, including numerical effects, such as findings that specific feedback and the effects of cleaning bundles, along with the effect of TSs, can help hospitals in developing IC programs. In light of these findings, we suggest that the PHC continue providing hospitals under its jurisdiction with effective TSs on IC programs for hospitals, including numerical effects. This will further support and facilitate the development of IC practices in these hospitals.
